# Knockdown of FABP3 Impairs Cardiac Development in Zebrafish through the Retinoic Acid Signaling Pathway

**DOI:** 10.3390/ijms140713826

**Published:** 2013-07-03

**Authors:** Xuejie Wang, Lijuan Zhou, Jin Jin, Yang Yang, Guixian Song, Yahui Shen, Hailang Liu, Ming Liu, Chunmei Shi, Lingmei Qian

**Affiliations:** 1 Department of Emergency, Subei People Hospital, Yangzhou, Jiangsu 225001, China; E-Mail: wangxuejie860830@163.com; 2 Department of Cardiology, the First Affiliated Hospital of Nanjing Medical University, Nanjing 210029, China; E-Mails: 913zhoulijuan@163.com (L.Z.); qtjinjin@163.com (J.J.); yangyang19861220@sina.com (Y.Y.); songguixian1983@163.com (G.S.); lhl2ny@yeah.net (H.L.); l158506@163.com (M.L.); 3 State Key Laboratory of Reproductive Medicine, Department of Pediatrics, Nanjing Maternity and Child Health Hospital Affiliated to Nanjing Medical University, Nanjing 210029, China; E-Mail: shenyahui1986@163.com

**Keywords:** FABP3, zebrafish, cardiac development, RA

## Abstract

Fatty acid-binding protein 3 (FABP3) is a member of the intracellular lipid-binding protein family, and is primarily expressed in cardiac muscle tissue. Previously, we found that FABP3 is highly expressed in patients with ventricular-septal defects and is often used as a plasma biomarker in idiopathic dilated cardiomyopathy, and may play a significant role in the development of these defects in humans. In the present study, we aimed to investigate the role of FABP3 in the embryonic development of the zebrafish heart, and specifically how morpholino (MO) mediated knockdown of FABP3 would affect heart development in this species. Our results revealed that knockdown of FABP3 caused significant impairment of cardiac development observed, including developmental delay, pericardial edema, a linear heart tube phenotype, incomplete cardiac loop formation, abnormal positioning of the ventricles and atria, downregulated expression of cardiac-specific markers and decreased heart rate. Mechanistically, our data showed that the retinoic acid (RA) catabolizing enzyme Cyp26a1 was upregulated in FABP3-MO zebrafish, as indicated by in situ hybridization and real-time PCR. On the other hand, the expression level of the RA synthesizing enzyme Raldh2 did not significantly change in FABP3-MO injected zebrafish. Collectively, our results indicated that FABP3 knockdown had significant effects on cardiac development, and that dysregulated RA signaling was one of the mechanisms underlying this effect. As a result, these studies identify FABP3 as a candidate gene underlying the etiology of congenital heart defects.

## 1. Introduction

The heart is the first organ to form and function during vertebrate embryonic development. Congenital heart disease is an important component of pediatric cardiovascular disease present at birth, and constitutes a major percentage of clinically significant birth defects, with an estimated prevalence of 4–50 per 1000 live births [1,2]. Congenital heart defects occur in almost 1% of live births, reflecting the complex cellular processes underlying heart development [3]. Cardiac development requires a precise and extremely complex series of molecular mechanisms to orchestrate the proper expression of cardiac transcription factors, and alterations in their expression can result in heart defects [4,5]. Classical forward genetic human pedigree studies have thus far provided limited insight into the genetic causes of congenital heart defects.

Previously, McCann *et al*. found that fatty acid-binding protein 3 (FABP3; also referred to as heart-type fatty acid-binding protein) could be used as a novel plasma biomarker in the early diagnosis of acute ischemic chest pain [6]. Earlier, our group also established that FABP3 is upregulated in patients with ventricular-septal defects in comparison to normal controls, by using subtractive hybridization [7]. We have also reported that down-regulation of FABP3 expression promotes cell apoptosis and causes mitochondrial dysfunction in P19 cells [8]. Interestingly, FABP3 has been shown to be upregulated during terminal differentiation of mouse cardiomyocytes [9]. Taken together these studies suggest that FABP3 may play an important role in cardiac development; however, definitive data is lacking.

One factor that is known to be important in cardiac development is retinoic acid (RA). For example, the size of the myocardial progenitor pool is restricted by RA signaling [10], and evidence from several studies has suggested that excess or reduced levels of RA can result in cardiac defects [10,11]. It is known that altered RA levels by regulated activation of the Raldh2 gene in avian embryos supports a role for RA signaling in development of the venous pole of the heart [12].

In recent years, the zebrafish has become one of the best vertebrate models for studying developmental biology. Cardiovascular development in the zebrafish is well defined and has been shown to be morphologically and physiologically similar to that of mammals [13,14]. Additionally, early-stage cardiac development of zebrafish is similar to that of the human heart in many respects, such as migration of cardiac precursor cells towards the central line, heart tube formation, early chamber formation and the looping process [13,15]. Thus, studies in the zebrafish embryo can facilitate the characterization of cardiac defects and their underlying molecular mechanisms.

As discussed above, FABP3 is a gene of interest in cardiac development; moreover, it has previously been established that FABP3 strong expressed in the central nervous system (CNS), retina and myotomes using situ hybridization, FABP3 mRNA is expressed in the developing zebrafish [16,17]. In this study we used a zebrafish model to investigate the role of FABP3 in cardiac development and dissect its underlying molecular mechanisms, particularly with regard to its interaction with the RA signaling pathway. To achieve this goal, we used a morpholino (MO) approach to silence FABP3 expression and then examined the resultant effect of FABP3 knockdown on cardiac development and heart function in zebrafish embryos. We also analyzed the alteration of RA signaling pathway to determine if FABP3 knockdown has an effect on this important pathway in cardiac development.

## 2. Results

### 2.1. Verification of FABP3 Knockdown by FABP3-MO Injection in Zebrafish

**Figure 1:**
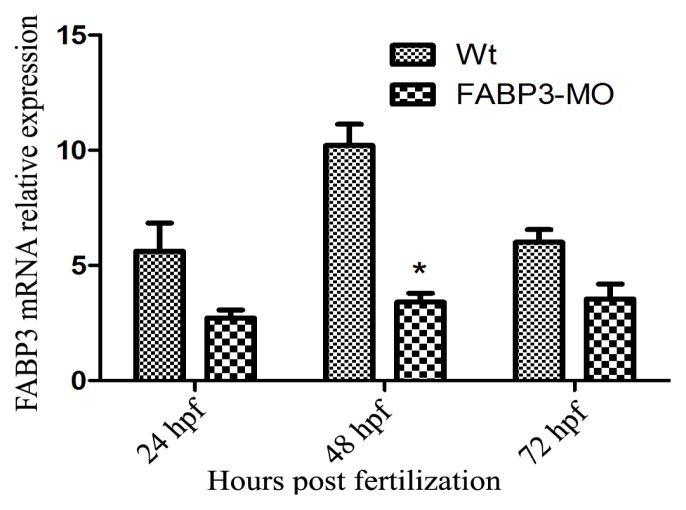
Expression of FABP3 is downregulated in FABP3-MO injected zebrafish. Quantitative PCR validation of FABP3 expression in FABP3-MO injected zebrafish at 24, 48 and 72 hpf and Wt-MO injected zebrafish. Data are representative of three independent experiments; data are expressed as the means ± SD; *n* = 50 zebrafish embryos per group. *p < 0.05.

To understand the function of FABP3 in zebrafish cardiac development, we used MO antisense technology; this method has previously been shown to reliably knock down target gene expression levels thereby preventing translation of the target gene in zebrafish [18]. We injected 5.0 ng of FABP3-MO and Wt-MO into single-to-four-cell stage zebrafish embryos, and then extracted total RNA at 24, 48 and 72 hpf. The inhibition efficiency of the FABP3-MO was assessed by real-time PCR; as shown in Figure 1 the expression of FABP3 was decreased in FABP3-MO injected embryos compared with Wt-MO embryos, at 24, 48 and 72 hpf. This effect was greatest at 48 hpf, when expression of FABP3 was decreased by more than 70% compared with Wt-MO (*p* < 0.05).

### 2.2. FABP3-MO Injection Was Associated with Zebrafish Embryo Lethality

The effect of FABP3-MO on mortality rate at different concentrations and under different developmental stages (24, 48, 72, 96, and 120 hpf) was determined. The percentage of embryos suffering acute toxicity at each treatment concentration was calculated. As shown in Table 1, the rate of lethality resulting from FABP3-MO at a dose of 1.0 ng up to 120 hpf was no more than 15% compared with Wt-MO and the non-injected control group. In contrast, the rates of lethality resulting from FABP3-MO dosages at 2.5 ng and 5.0 ng were 22.0% and 37.7% at 120 hpf, respectively. When embryos were treated with 7.5 ng and 10.0 ng FABP3-MO, the rate of lethality exceeded 70% and 97%, respectively, making it impossible to observe heart development during later stages of development. Additionally, when higher concentrations of FABP3-MO were coupled with longer observation times, the rate of lethality among treated embryos increased substantially. Based on our systematic investigation of MO dose and the resultant effect on embryo lethality, we injected FABP3-MO at a dosage of 5.0 ng to investigate the influence of FABP3 on cardiac development.

### 2.3. FABP3-MO Causes Zebrafish Embryonic Malformation, Especially in the Heart

**Figure 2:**
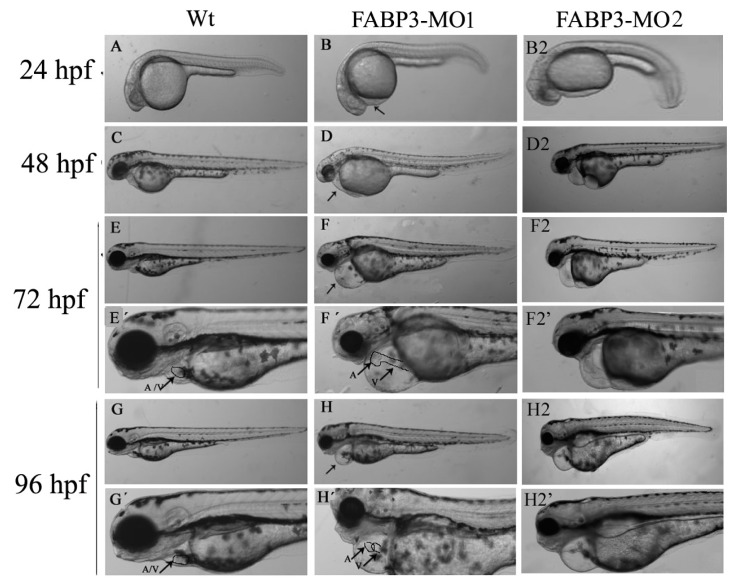
Microinjection of FABP3-MO induced abnormal embryonic development, particularly in the heart. Representative images of zebrafish embryos are shown. Compared to the Wt-MO group (**A**), embryos injected with FABP3-MO at 24 hpf (**B**, **B2**) displayed slightly pericardial edema (black arrows and developmental delay). At 48 hpf, FABP3-MO1 and FABP3-MO2 injected zebrafish embryos (**D** and **D2**) displayed phenotypes similar to the embryos treated at 24 hpf, except with abnormal somites and head malformations which were not seen in age-matched control embryos (**C**). These defects appeared more severe at 72 hpf in the FABP3-MO1 and FABP3-MO2 group (**F**, **F2**) compared to the control group (**E**). Similarly, severe abnormalities were observed in FABP3-MO1 and FABP3-MO2 injected embryos at 96 hpf (**H**, **H2**), but not control embryos (**G**). Higher magnification (630 multiple) images of embryos are shown in part 2 at 72 and 96 hpf. FABP3-MO injected embryos show overlapping of the ventricle and atrium, and a linear heart tube at 72 hpf (**F**′, **F2**′), which was not evident in control embryos (**E**′). Similarly, the ventricle and atrium could be viewed at the same position at 96 hpf embryos in the FABP3-MO group (**H**′, **H2**′), but not in the control group (**G**′). V: ventricle; A: atrium.

Embryos were microinjected with 5.0 ng FABP3-MO1 (against the ATG start site) to observe the effect of FABP3 knockdown on zebrafish development, with a particular focus on heart development. In contrast with Wt-MO injected zebrafish, embryos injected with FABP3-MO1 at 24 hpf had slight pericardial edema and developmental delay (Figure 2A,B). When we examined embryos at 48 hpf, the embryos showed heart phenotypes similar to those at 24 hpf, but slightly abnormal somites and head malformations were also observed (Figure 2C,D). With longer observation times (72 hpf), pericardial edema was more severe, and we also noted the occurrence of smaller heads and abnormal eye development (Figure 2E,F). However, these embryos were smaller and showed pericardial edema, developmental delay, smaller heads and abnormal eye development at 96 hpf compared with 24, 48, 72 hpf (Figure 2H,G). In general, we observed that FABP3-MO1 injected embryos were characterized by pericardial edema and developmental delay.

Normally, cardiac looping starts around 36 hpf, with a displacement of the ventricle towards the mid-line, and the constriction at the position of the atrioventricular canal is first visible. The linear heart tube bends towards the right side after it has formed. This bending is best described as a displacement of the future ventricle so that it will be positioned at the right side of the embryonic mid-line, while the future atrium remains positioned at the left side of the mid-line. Heart tubes that extend to the right side of the embryo will loop ultimately in the reverse direction [19]. By 48 hpf, posterior circulation formation has completed, thus, we chose 72 and 96 hpf to further examine the pericardial edema observed in FABP3-MO1 embryos. As shown in Figure 2, high-magnification imaging of FABP3-MO1 embryos revealed a linear heart tube phenotype at 72 hpf, as well as incomplete looping of the developing heart (Figure 2E′,F′). At 96 hpf, we can see the position of ventricle and atrium overlapped in FABP3-MO of embryos from side view (Figure 2G′,H′). Taken together, these results indicate that FABP3-MO injection caused abnormal heart tube development in zebrafish embryos.

Furthermore, to minimize off-target effects and strengthen the impact of the observed heart phenotype, Eisen *et al*. recommended that it is a must to design at least two morpholinos against the target gene. Thus, we designed the splice-Morpholino of FABP3 (FABP3-MO2) and observed the heart phenotype. We can see these zebrafish development and heart development phenotype were the same as FABP3-MO1 induced abnormal development phenotype, which strengthen the fact that knockdown of FABP3 induced heart phenotype is not an off-target effect. Thus, we used FABP3-MO1 to investigate the influence of FABP3 on cardiac development in the follow-up study.

### 2.4. Decreased Heart Rate in FABP3-MO Injected Embryos

**Figure 3:**
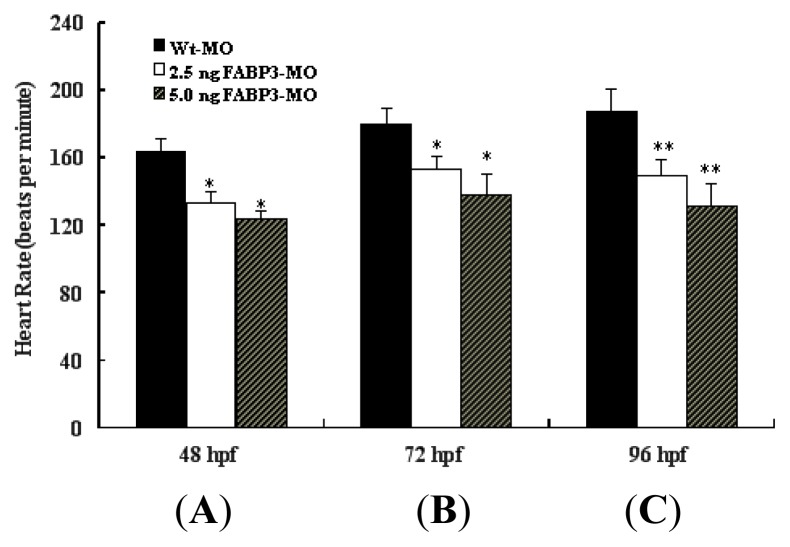
FABP3-MO decreased heart rate in zebrafish embryos. Heart rate, measured in bpm, was counted in embryos injected with 2.5 and 5.0 ng FABP3-MO at 48, 72 and 96 hpf. The heart rate decreased in FABP3-MO injected embryos, compared to Wt-MO at 48 (**A**), 72 (**B**) and 96 hpf (**C**). Note that the heartbeat was gradually elevated with increasing age in Wt-MO and FABP3-MO at the three time points (**A**–**C**). * *p* < 0.05; ** *p* < 0.01.

In addition to our morphological observation of the developing heart, we also measured the heart rate in FABP3-MO treated embryos. The heart rate was examined at 48, 72 and 96 hpf. As shown in Figure 3A,B, the mean heart rate of FABP3-MO injected embryos at a dosage of 2.5 and 5.0 ng, was significantly lower than that in Wt-MO injected embryos at 48 and 72 hpf (*p* < 0.05). The ventricles and atria of Wt-MO injected embryos exhibited vigorous, rhythmic contractions, ensuring circulation throughout the body at 72 hpf. At 96 hpf, the average heart rate of Wt-MO injected embryos was 187.1 ± 12.9 beats per minute (*n* = 10), whereas the heart rate of FABP3-MO injected embryos at a dosage of 2.5 and 5.0 ng was 148.7 ± 9.5 (*n* = 10) and 130.8 ± 13.4 (*n* = 10) beats per minute, respectively. These heart rate values were significantly lower in the FABP3-MO injected embryos compared to Wt-MO injected embryos at 96 hpf (*p* < 0.01; Figure 3C). Interestingly, we noted that heart rate increased in Wt-MO injected embryos at 96 hpf when compared with that at 48 and 72 hpf; however, from 48 to 96 hpf (Figure 3A–C), the trajectory of this increase was significantly different between Wt-MO (*p* < 0.01) and FABP3-MO (*p* < 0.05) injected embryos.

### 2.5. Effect of FABP3-MO on the Expression of Cardiac-Specific Genes in Zebrafish

**Figure 4:**
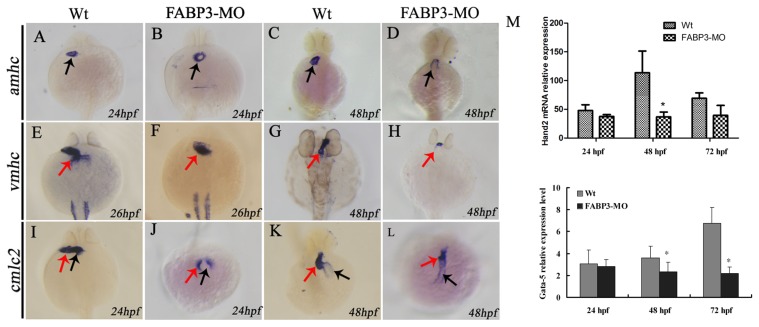
Effect of FABP3-MO on the expression of cardiac-specific genes in zebrafish. Cardiac-specific markers amhc (24 and 48 hpf), vmhc (26 and 48 hpf) and cmlc2 (24 and 48 hpf) were used to observe cardiac morphology within the whole embryo using *in situ* hybridization (red arrows), and the expression of Hand2 was assayed by real-time PCR at 24, 48 and 72 hpf, respectively. Compared to Wt-MO (**A**), amhc expression was abnormally positioned (**B**) at 24 hpf, and was significantly downregulated in FABP3-MO injected embryos at 48 hpf (**D**), as compared to control embryos (**C**). Vmhc expression was also in an abnormal position and appeared lower in FABP3-MO treated embryos at 24 hpf (**F**) and 48 hpf (**H**), compared to control embryos at 24 hpf (**E**) and 48 hpf (**G**), respectively. Cmcl2 expression revealed that heart looping was incomplete and formed a liner cardiac tube in FABP3-MO injected embryos (**K**), compared to the normally looped heart in control embryos (**K**). Hand2 and Gata5 expression is downregulated in zebrafish induced by FABP3-MO compared to Wt-MO at the three time points examined (**M**). * *p* < 0.05.

To further investigate the effect of FABP3-MO on cardiac development, we next determined the expression level of cardiac development-specific genes including amhc (at 24 and 48 hpf), which is expessed in the developing atrium; vmhc (at 26 hpf and 48 hpf), which is expressed in ventricular cells; and cmlc2 (at 24 and 48 hpf), which is initially expressed only in a few heart cells, but increases over time [20]. Expression of these genes was determined at the tissue level during embryonic development by whole embryo in situ hybridization. Embryos microinjected with 5.0 ng FABP3-MO revealed that amhc was abnormally expressed in the atria, compared to control embryos at 24 hpf (Figure 4A,B), and appeared to be downregulated at 48 hpf (Figure 4C,D), resulting in abnormal atrial developmental position (Figure 4B,D). As shown in Figure 4E,F, vmhc expression was abnormally distributed at 26 hpf and appeared reduced in FABP3-MO embryos compared to Wt-MO embryos. By 48 hpf, these alterations were more severe and vmhc expression was significantly reduced (Figure 4G,H). The expression pattern of cmlc2 was also found to be abnormal in FABP3-MO injected embryos, compared to Wt-MO at 24 hpf (Figure 4I,J). While at 48 hpf, incomplete looping of the developing heart was observed in FABP3-MO injected embryos forming a linear cardiac tube (Figure 4L), and in Wt-MO embryos the heart tubes extended to the right side of the embryo and ultimately looped in the normal direction (Figure 4K).

Finally, we examined the expression level of Hand2 and Gata5, which are required for cardiogenic differentiation. Interestingly, our real-time PCR results revealed that Hand2 and Gata5 were downregulated in zebrafish embryos injected with FABP3-MO compared to Wt-MO, particularly at 48 hpf, when the expression level of cardiogenic differentiation key gene were significantly decreased in FABP3-MO injected zebrafish and Gata5 was decreased in FABP3-MO injected zebrafish at 72 hpf (Figure 4M). Thus, knockdown of FABP3 not only induces significant toxic effects on cardiac development but these effects are also associated with downregulated expression of Gata5, which interact to induce Hand2 expression, which influence looping of the developing heart. This data indicates that FABP3 may be an upstream regulator of important heart genes Gata5 and Hand2 involved in looping of the heart in zebrafish embryos.

### 2.6. FABP3-MO Alters the Retinoic Acid Signaling Pathway in Zebrafish Embryos

**Figure 5:**
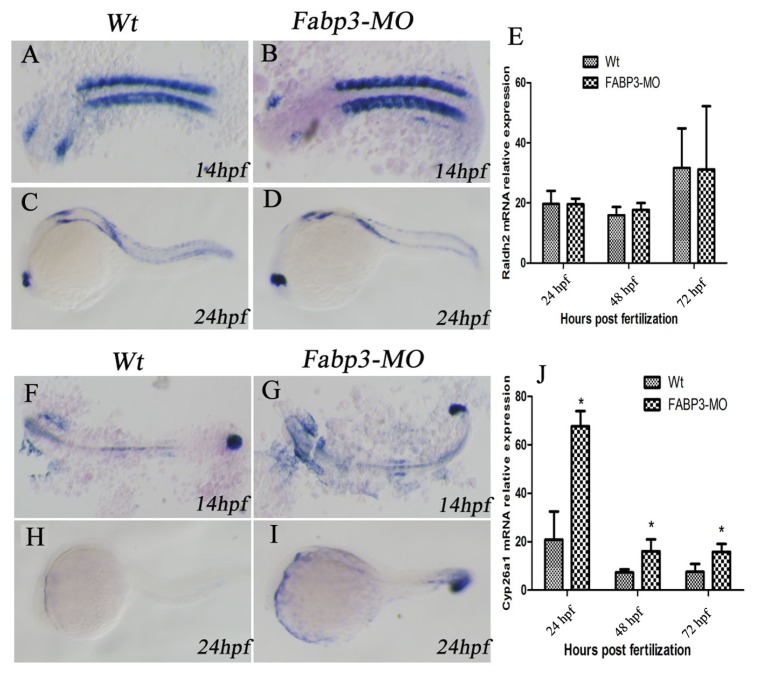
FABP3-MO alters the retinoic acid signaling pathway. Raldh2 expression was not significantly different in FABP3-MO injected zebrafish, as visualized by *in situ* hybridization at 14 hpf (**B**) and 24 hpf (**D**), compared with Wt controls at 14 hpf (**A**) and 24 hpf (**C**), respectively. Stable expression levels of Raldh2 were also confirmed by real-time PCR (**E**). Cyp26a1 expression was significantly upregulated in FABP3-MO injected zebrafish, as visualized by in situ hybridization at 14 hpf (**G**) and 24 hpf (**I**), compared with Wt controls at 14 hpf (**F**) and 24 hpf (**H**), respectively. Increased expression levels of Cyp26a1 were confirmed by real-time PCR (**J**).* *p* < 0.05.

We next investigated the mechanism through which FABP3 knockdown could affect cardiac development by focusing on the RA signaling pathway. RA is a small lipophilic molecule derived from Vitamin A and is regarded as the first developmental morphogen identified in vertebrates [21]. We chose two time points (14 and 24 hpf) to detect changes in the expression of Raldh2 and Cyp26a1 using situ hybridization in FABP3-MO embryos, with supporting experiments carried out by real-time PCR at 24, 48 and 72 hpf. As shown in Figure 5A–D, the expression pattern of Raldh2 was not significantly different at the 14 and 24 hpf time points in FABP3-MO injected zebrafish, and mRNA expression levels were also not significantly different at 24, 48 and 72 hpf. Conversely, we found that there was enhanced Cyp26a1 expression evident at 14 and 24 hpf (Figure 5F–I), which was confirmed to be statistically significant at the mRNA expression level at 24, 48 and 72 hpf (Figure 5J). These results indicated that FABP3-MO influenced the RA signaling pathway via up-regulation of Cyp26a1, which may also have contributed to abnormal cardiac development.

## 3. Discussion

In the last decade, zebrafish have been used as a powerful model to study cardiac development [13]. The mechanisms of zebrafish development are thought to be similar to mammals, thus an improved understanding of zebrafish development can be related to developmental mechanisms in these animals. The zebrafish embryonic attributes augment the feasibility of conducting classical genetic screens that affect cardiac development. Thus, we used zebrafish as a model to investigate the FABP3 in cardiac development. We have previously reported that FABP3 is highly expressed in patients with ventricular-septal defects, when compared with normal controls [7]. This and other observations led us to hypothesize that FABP3 might play an important role in cardiac development. In the present study, cardiac development was explored in FABP3-MO injected zebrafish embryos and the resultant zebrafish heart defects were further studied at the molecular level.

After establishing that our FABP3-MO led to knockdown of FABP3 in zebrafish embryos, we performed a systematic investigation of zebrafish heart development. We found that injection of FABP3-MO substantially increased the rate of embryonic lethality in a dose-dependent manner. Based on these preliminary studies, a dose of 5.0 ng of FABP3-MO was chosen to study the effect of FABP3 knockdown in heart development. To minimize off-target effects and strengthen the impact of the observed heart phenotype, we designed the splice-Morpholino of FABP3 (FABP3-MO2) and observed the heart phenotype. At a dose of 5.0 ng MO, we found embryonic or larvae malformations, developmental delay, smaller heads, abnormal eye development, pericardial edema and a linear heart tube phenotype in FABP3-MO injected zebrafish. We interpreted these results to indicate that FABP3 can influence the development of zebrafish, especially with regard to the heart.

It is known that when the bilateral heart fields fuse at the mid-line, they form a cardiac disc structure with the endocardial cells within a hole at the center, atrial myocytes at the periphery and ventricular myocytes around the circumference of the disc [15]. After heart tube fusion, the heart begins to slowly beat at about 25 times/min at 22 hpf, then the heartbeat gradually increases to about 90 times/min. Interestingly, in addition to the structural defects described above, decreased heart rate was also detected in FABP3-MO injected embryos; however, the trajectory of heart rate was increased in FABP3-MO injected embryos from 48 to 96 hpf (Figure 3). This data confirmed that FABP3-MO injection impaired heart development, which was reflected in decreased heart function.

The myocardial cell population of the developing heart is polarized in a medial to lateral direction, with the medial cells expressing vmhc and the lateral cells expressing amhc. Expression of amhc is known to be initiated slightly later compared than the onset of expression of vmhc [22], which suggested that the differentiation of these two myocardial cell types is initiated at different time points. Expression of cmlc2 is initiated in a few cells, with the number of cells expressing cmlc2 increasing over time [20]. This strict organization and separation of ventricular and atrial myocytes is maintained during the later stages of cardiac development, although it is currently unclear by what mechanism these two cell populations are kept separated. Given the importance of these factors in heart development, we analyzed their expression level in FABP3-MO injected embryonic zebrafish, with our results showing that amhc and vmhc expression was downregulated. Furthermore, our cmlc2 expression data indicated that heart looping was incomplete in FABP3-MO injected zebrafish, which was consistent with our morphological examination of the developing heart. Taken together, our data strongly indicated that FABP3-MO injection impaired cardiac development in zebrafish embryos, and that these hearts underwent abnormal heart looping.

It is known that Hand2 and Gata5 are required for cardiogenic differentiation, because hands off/hand2 mutant embryos have dramatically reduced myocardial tissue [23]. In addition, Gata5 interact to induce Hand2 expression. It has also been established that Hand2 also has a non-cell-autonomous role during fusion of the bilateral cardiac fields by repressing fibronectin production [24]. Our results showed that Hand2 and Gata5 are downregulated in zebrafish injected with FABP3-MO at 48 and 72 hpf, and this time point was also the FABP3 expression level decreased when zebrafish injected with FABP3-MO, indicating that FABP3 may be an upstream regulator of Gata5 and Hand2, and that the heart defects observed in FABP3-MO injected embryos may have their origin in dysregulated Gata5 and Hand2 expression. However, the time course of FABP3 affected betweenGata5 and Hand2 expression was still unknown, which needs further study.

In addition to the important heart genes, we were also interested in examining other signaling pathways that are known to be important in heart development. In this regard, it is known that multiple signaling pathways converge to regulate heart development, including RA, Wnt, BMP, and Hh signaling [25]. In this study, we focused on the RA signaling pathway in FABP3-MO injected zebrafish. Our results indicated that the RA-metabolizing enzyme, Cyp26a1 was upregulated in FABP3-MO injected embryos, though there was no significant effect on the expression of Raldh2, which synthesizes RA. It is known that RA can regulate the size of the cardiac field in zebrafish [10,12], and that excess or reduced RA levels can result in cardiac defects. For example, altered RA levels in avian embryos supports a role for RA signaling in development of the venous pole of the heart [12]. It has also recently been suggested that RA signaling may participate in defining the posterior limit of the second heart field formation, heart tube fails to extend in Raldh2 mutant embryos [11,26]. Interestingly, knockout of Raldh2 in mouse embryos reveals that a number of second heart field formation genes are abnormally expanded in a posterior direction, including Isl1, Tbx1, Fgf10, and Fgf8 [11,26]. Besides, retinoic acid signaling is in turn controlled by signals from the second heart field: Cyp26 genes a1, b1 and c1 are down-regulated in Tbx1 null mice and inhibition of Cyp26 enzyme function [27] Our results indicated that FABP3-MO influences the RA signaling pathway through altered Cyp26a1 expression, which could lead to cardiac developmental abnormalities by dysregulating RA levels in the developing heart.

## 4. Experimental Section

### 4.1. Embryo Maintenance and Transplantation

Wild type zebrafish stocks were obtained from the Model Animal Research Center of Nanjing University. Embryos were obtained from natural spawning of wild type adults, and were grown at 28.5 °C in embryo medium as previously described [28]. Morphological features were used to determine the embryonic developmental stage, as described by Kimmel *et al*. [29]. Embryos older than 24 h post-fertilization (hpf) were incubated in 0.003% phenylthiourea to inhibit pigment formation.

### 4.2. Morpholinos and Microinjection

MOs (morpholinos) were purchased from Gene Tools (Philomath, OR, USA). The FABP3 MO was designed against the ATG start site of FABP3 to block its translation (FABP3-MO1; 5′-ACG TGC CGA TAA AAG CGT CTG CCA T-3′), and we also designed the oligo to target the exon 2/intron 2 splice boundary by Gene Tools (FABP3-MO2; 5′-AGC CAA CAC CTG ATA AAG CAA ACA T-3′). A standard control MO (Wt-MO; 5′-CCT CTT ACC TCA GTT ACA ATT TAT A-3′) served as negative control. MO oligomers were dissolved in 0.3 × Danieaus’s solution (58 mmol/L NaCl, 0.7 mmol/L KCl, 0.4 mmol/L MgSO_4_, 0.6 mmol/L Ca(NO_3_)_2_, 5.0 mmol/L *N*-[2-Hydroxyethyl] piperazine-*N*′-[2-ethanesulfonic acid] (HEPES), pH7.6) at 2 mmol/L [20], and diluted to their final concentration in 0.2 mol/L KCl and 0.5% (*w*/*v*) phenol red (Sigma, St. Louis, MO, USA). Injection of 1.0, 2.5, 5.0, 7.5 and 10.0 ng of MO into single to four-cell stage zebrafish embryos using back-filled fine borosilicate glass capillary needles was performed as previously described [18]. Following MO injection, embryos were incubated at 28.5 °C for up to 3 days post-fertilization.

### 4.3. Determining the Rate of Lethality and Observation of Zebrafish Heart Development

After microinjection of MOs into zebrafish embryos, we counted the rates of lethality and identified heart-specific phenotypes and malformations using an Olympus SZ61 dissecting microscope (Olympus, Tokyo, Japan) at the following time points: 24, 48, 72 and 96 hpf. Embryos were photographed with a DP70 digital camera (Olympus), and images were processed using Adobe Photoshop software (Adobe Systems, San Jose, CA, USA).

### 4.4. Heart Rate Measurement

Embryos were anesthetized in ethyl aminoboenzoate (Tricaine, Sigma, St. Louis, MO, USA). A Tricaine solution stock was made as follows: 400 mg of Tricaine powder, 97.9 mL ddH_2_O and 2.1 mL 1 mol/L Tris (pH 9). The solution was adjusted to pH 7 and stored in the freezer until required for further use. To anesthetize fish, 4.2 mL of Tricaine solution stock was diluted in a clean 100 mL water tank. Anesthetized embryos were then transferred to a recording chamber perfused with modified Tyrode’s solution (136 mmol/L NaCl, 5.4 mmol/L KCl, 0.3 mmol/L NaH_2_PO_4_, 1.8 mmol/L CaCl_2_, 1 mmol/L MgCl_2_, 10 mmol/L HEPES, 5 mmol/L glucose, pH 7.3) at 48 hpf. The heart rate was calculated by counting the number of sequential contractions, beginning and ending at the end diastole. This was conducted under a dissecting microscope in 30 s intervals.

### 4.5. Preparation of RNA Probes

RNA Probes used for in situ hybridization included: atrial myosin heavy chain (amhc), ventricula rmyosin heavy chain (vmhc), cardiacmyosin light chain-2 (cmlc2), retinaldehyde dehydrogenase gene (Raldh2) and Cytochrome P450 26A1 (Cyp26a1). Probes were amplified from cDNA generated from embryos at 48 hpf and subcloned into pBluescript-KS vector (Stratagene, La Jolla, CA, USA). These plasmids were then linearized and transcribed as follows: amhc, SalI (Takara, Osaka, Japan)/SP6; vmhc and cmlc2, SalI/SP7; Raldh2 and Cyp26a1, NcoI (Takara, Osaka, Japan))/SP6. Each RNA probe was transcribed using a MAXIscript^®^*In Vitro* Transcription Kit (Invitrogen, Carlsbad, CA, USA). Digoxigenin-labeled single strand RNA probes were prepared using a digoxigenin RNA labeling kit (Roche Diagnostics, Basle, Switzerland) according to the manufacturer’s instructions, and stored at −40 °C for future use. For each gene, a sense (forward) probe was used as control, but was found to yield no specific signal.

### 4.6. Whole-Mount *in Situ* Hybridization

RNA in situ hybridization was performed as previously described [30] using riboprobes specific for vmhc, amhc, cmlc2, Raldh2 and Cyp26a1 [31]. To perform whole-mount in situ hybridization, we fixed the embryos overnight with PBS-based 4% paraformaldehyde at 4 °C. The following day, embryos were dechorionated manually and then transferred to 1.5 mL Eppendorf tubes. The embryos were dehydrated in a graded series of 50% methanol/PBS, 75% methanol/PBS and 100% methanol for 5 min, respectively. The 100% methanol was changed twice at 5 min intervals, and then the embryos were stored at −20 °C for at least 3 h until required for further processing. All further steps were performed at room temperature unless stated otherwise. To continue the experiment, the embryos were rehydrated by incubating for 5 min each in 50% methanol in PBST (PBS/0.1% Tween-20), 25% methanol in PBST, and twice in PBST. Embryos were then incubated at 65 °C for 5 min in tubes containing Hyb-solution (50% formamide, 5× SSC, 0.1% Tween-20). The Hyb-solution was replaced by Hyb+ (Hyb− with 5 mg/mL yeast RNA [Sigma, St. Louis, MO, USA]; and 50 mg/mL heparin [Sigma]) and the embryos were prehybridized in Hyb+ at 65 °C for 4 h up to 24 h. As much of the Hyb+ solution as possible was removed without allowing the embryos to come in contact with air, before 100 μL of fresh Hyb+ containing 50 to 100 ng of digoxigenin-labeled RNA probe (heated to 65 °C for 10 min then on ice 2 min prior to addition) was added to the tubes. After an overnight incubation at 65 °C the probe was removed and the embryos were washed twice for 30 min in 50% formamide/2 × SSC at 65 °C, followed by washing with 2× SSC (15 min at 65 °C) and two washes with 0.2× SSCT (30 min at 65 °C). Then embryos were blocked for 1 h in blocking buffer (2% blocking reagent [Roche, Indianapolis, IN, USA], 10% sheep serum and 70% Maleic acid (MAB) [Sigma, St. Louis, MO, USA]). Alkaline-phosphatase conjugated anti-digoxigenin fab-fragments (1:5000, Roche) were added in fresh blocking buffer. After overnight incubation, embryos were washed 8 times with MABT (MAB/0.1% Tween-20) (45 min each). Then, the overnight MABT washing solution was changed with equilibration buffer (100 mM Tris pH 9.5, 50 mM MgCl_2_, 100 mM NaCl, 1 mM levamisol [Sigma], and 0.1% Tween-20), and incubated with the buffer for 10 min. Detection was performed with new equilibration buffer containing the substrate NBT/BCIP solution. The reaction was monitored under a dissecting microscope and stopped when the hybridization signal was satisfactory. Stained specimens were washed in PBST for 5 min, dehydrated in methanol twice for 10 min each and mounted in 2:1 benzyl benzoate: benzyl alcohol (Sigma). Photomicrographs were taken using an Olympus DP71 digital camera (Olympus), and digital images were further processed for brightness and contrast with Adobe Photoshop software (version 7.0; Adobe Systems: San Jose, CA, USA).

### 4.7. Real-Time Polymerase Chain Reaction (PCR) for the Assessment of Cardiac-Specific Gene Expression

Total RNA was extracted from zebrafish embryos using TRIzol reagent (Invitrogen, Carlsbad, CA, USA), and the extracted RNA was quantified using a NanoDrop 2.0 spectrophotometer (Thermo Scientific; Waltham, MA, USA). Complimentary DNA was synthesized from 2 μg of total RNA by using an AMV Reverse Transcriptase Kit (Promega A3500; Promega, Madison, WI, USA) according to the manufacturer’s instructions. Real-time qPCR was performed using SYBR Green PCR Master Mix (Applied Biosystems, Foster City, CA, USA) on an Applied Biosystems 7500 Sequence Detection System (ABI 7500 SDS). Real-time PCR reaction conditions were 95 °C for 10 min followed by 40 cycles of 94 °C for 20 s, 60 °C for 20 s, and 72 °C for 30 s with a final extension of 7 min at 72 °C. FABP3, helix-loop-helix transcription factor (Hand2), Gata5, Raldh2 and Cyp26a1 expression was normalized against β-actin using the comparative CT method to determine the relative changes in mRNA expression in the target sample. The sequences of the primers used for each gene are shown in the Table 2.

### 4.8. Statistical Analysis

All values for statistical significance represent the mean ± standard deviation (SD). Statistical analyses were performed using the chi-squared test for categorical data (SPSS version 16.0; IBM Corporation: Armonk, NY, USA); dose-response relationships were analyzed by the Mantel–Haenszel test. Differences were considered significant for *p* < 0.05. Means and standard deviations were determined for at least three independent experimental replicates.

## 5. Conclusions

In summary, we have found that knockdown of FABP3 causes defects in zebrafish embryonic development, including developmental delay, pericardial edema, and a linear heart tube phenotype. Knockdown of FABP3 had particularly significant toxic effects on cardiac development, such as incomplete heart looping and abnormal positioning of the ventricles and atria, which was accompanied by downregulated expression of cardiac-specific genes. Our mechanistic data indicated that the underlying molecular mechanism for these defects could be associated with dysregulated RA signaling. We conclude that knockdown of FABP3 has toxic effects on zebrafish embryogenesis, impeding normal heart development through the RA signaling pathway. Further analysis of the relationship between RA signaling and cardiac-specific transcription factor expression may clarify the mechanism which leads to abnormal heart development in embryos with decreased FABP3 expression.

## Figures and Tables

**Table 1:** The mortality rate of zebrafish embryos injected with FABP3-MO.

Concentration (ng)		Treatment duration				

		24 hpf	48 hpf	72 hpf	96 hpf	120 hpf
Non-injection		12(4)	13(4.3)	13(4.3)	15(5)	15(5)

*Wt-MO*		16(5.1)	19(6.3)	20(6.7)	20(6.7)	20(6.7)

*FABP3-MO*	1.0	19(6.7) *	26(8.7) *	30(10) *	34(11.3) *	35(11.7) *
	2.5	31(10.3) *	50(16.7) *	59(19.7) *	66(22) *	66(22) *
	5.0	55(18.3) *	78(26) *	89(29.7) *	101(33.7) *	113(37.7) *
	7.5	92(30.7) *	123(41.1) *	157(52.1) *	181(60.1) *	215(71.7) *
	10.0	193(64.3) *	248(82.7) *	275(91.7) *	289(96.3) *	289(96.3) *

χ^2^		533.145	720.105	945.243	917.345	981.454

*p*		<0.001	<0.001	<0.001	<0.001	<0.001

χ trend		398.827	568.573	724.707	781.940	860.933

*P*		<0.001	<0.001	<0.001	<0.001	<0.001

All data are provided as the number of embryos with the percent total in parenthesis; the total number of embryos examined = 300.

* indicates a significant difference between FABP3-MO injected embryos compared with Wt-MO and Non-injected embryos (*p* < 0.05).

**Table 2:** Sequences for primer sets used in qPCR gene expression analysis.

Gene name	Sense (5′→3′)	Antisense (5′→3′)
FABP3	CGATGAGTACATGAAAGGAA	GTGGATTTGAAAGTGCTGAC
Hand2	TACCATGGCACCTTCGTACA	CCTTTCTTCTTTGGCGTCTG
Gata5	CCACCGAATTCTGATCCGAGACC	GGAGGCTCGAGAAACGATATAATTCC
Cyp26a1	ACCATCGTGCTACCCGTTTT	GGCGGTAGAGGACTTCTGCA
Raldh2	CATTTTTGCAGATGCTGATTTTG	CAAAGATACGGGAACCAGCAGT
β-atcin	CAACAGAGAGAAGATGACACAGATCA	GTCACACCATCACCAGAGTCCATCAC
